# Supplementary material for: The Bovine Seminal Plasma Protein PDC-109 Possesses Pan-Antiviral Activity
Source: Viruses. 2022 Sep 13;14(9):2031. doi: 10.3390/v14092031 (PMC9504757; doi:10.3390/v14092031)
Supplement: Supplementary file 1 [file viruses-14-02031-s001.zip › viruses-1902510-SI.pdf]

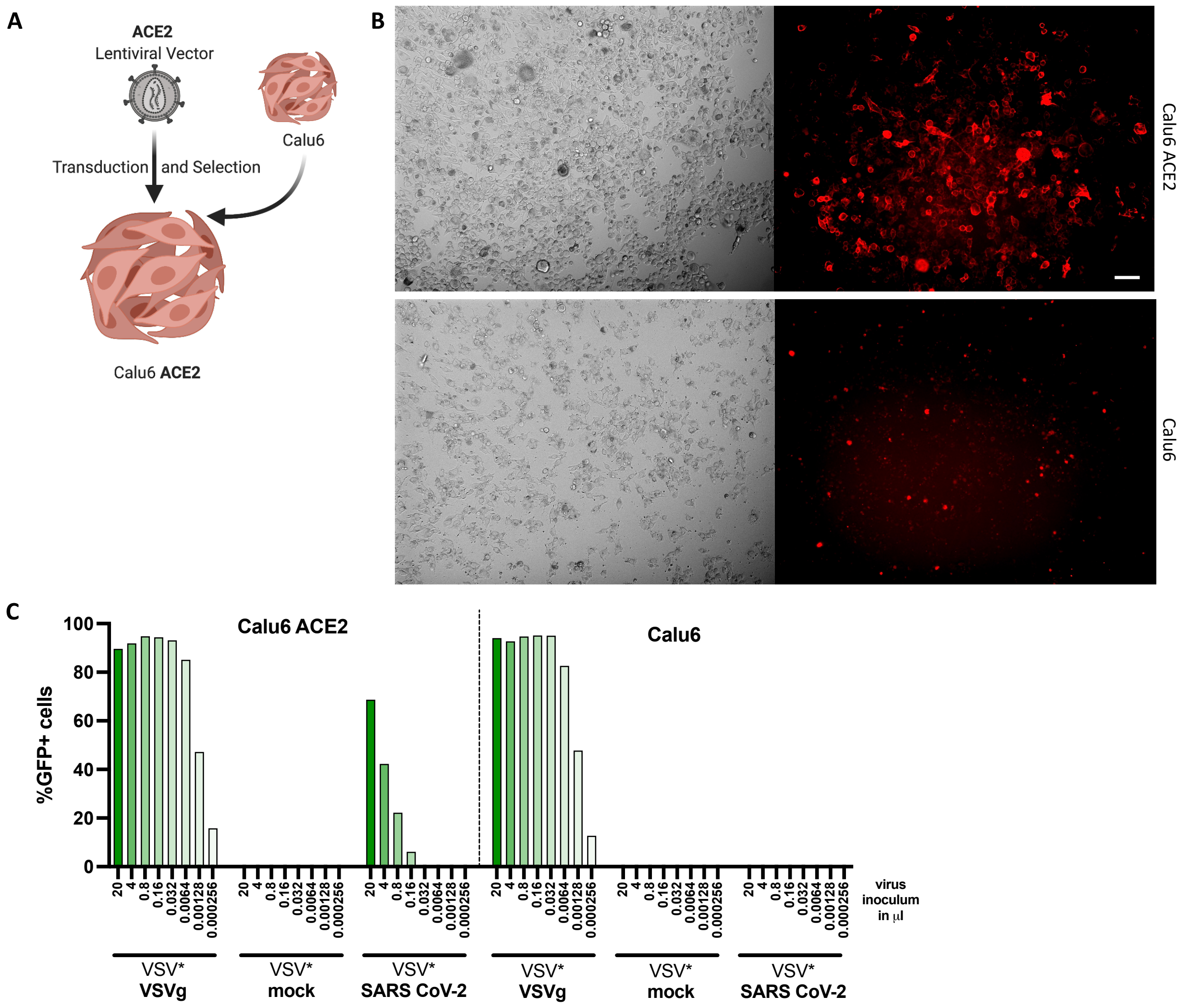

Figure S1: Generation of Calu6 ACE2 cells and SARS CoV-2 pseudotyping. (A) *The human lung-derived epithelial cell line Calu6 was transduced with lentiviral particles encoding for human ACE2 (hACE2) and a puromycin resistance cassette. After sufficient selection with Puromycin, a stable sub-cell line was obtained, henceforward termed Calu6 ACE2. (B) Phase constrast (left) and Immunofluorescence staining of Calu6 and Calu6 ACE2 cells with fluorescently tagged ACE2 antibodies (right). Micrographs were acquired using a Keyence BZ-X700 fluorescence microscope, using a 10x air (10x/0.30NA Ph1 DL) objective. Scale bar 100 µm.* ***(C)*** *Testing of VSV* SARS CoV-2 virus particles. Both cell lines were infected for 24 h with increasing concentrations of VSV* SARS mock (no pseudotype), VSV* SARS CoV-2 and VSV* VSVg, respectively. Bar charts show frequencies of GFP positive, live cells assessed by flow cytometry.*

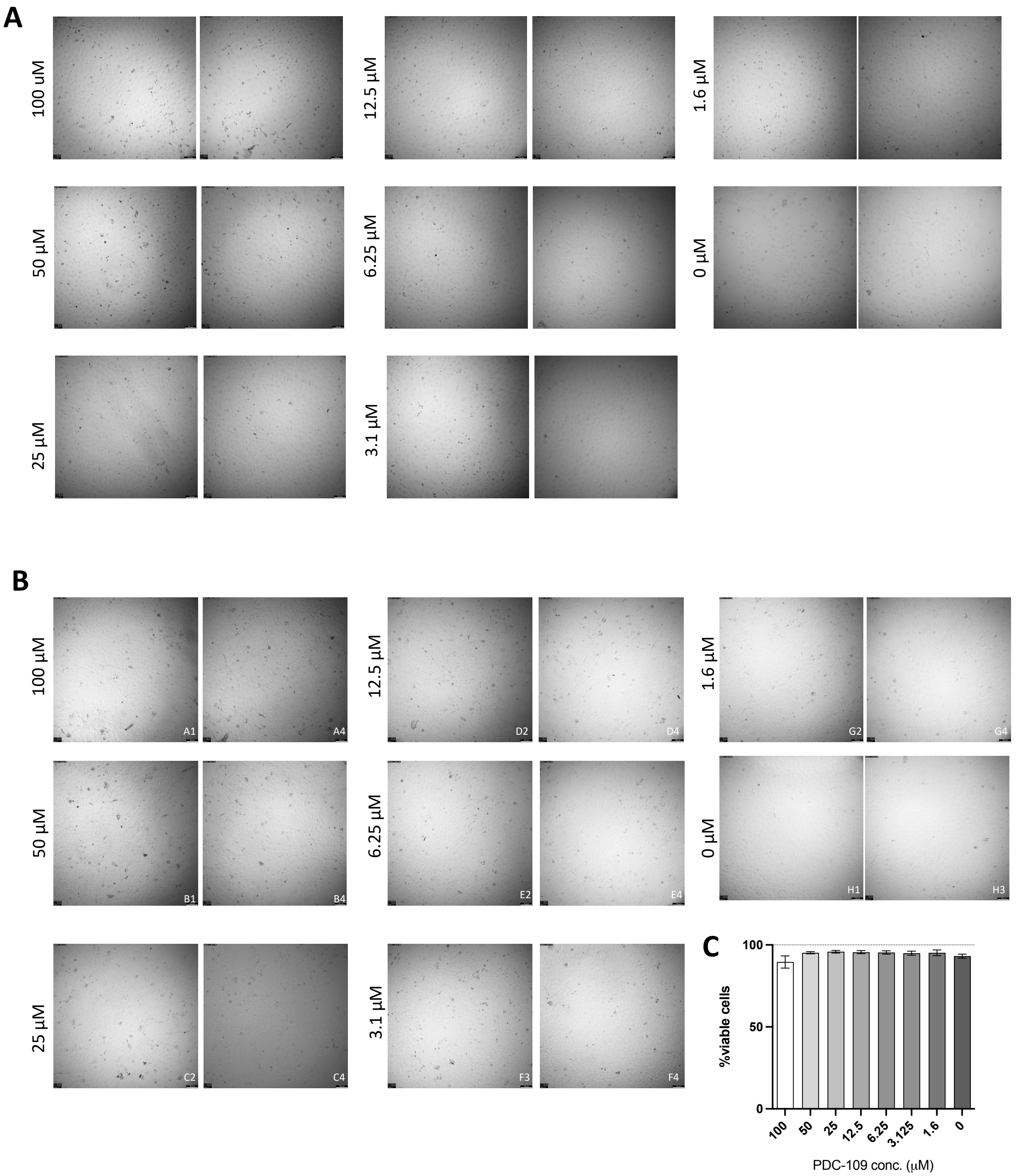

Figure S2: Cytotoxicity of PDC-109 in VeroE6 cells. *Cells were plated in flat-bottom 96 well plates and at the next day treated with increasing concentrations PDC-109 for 2 hours. Subsequently, supernatants were replaced with fresh media and cells were cultured for another* ***(A)*** *24 or* ***(B)*** *72 hours. Then, cells were imaged using a Thunder Imaging System (Leica, Wetzlar, Germany) in order to assess cell morphology and overall confluence of the monolayer.* ***(C)*** *Cells treated as described in (B) were subjected to cell viability staining using Zombie-Aqua (BioLegend, San Diego, USA), followed by flow cytometry at a LSRII instrument. Cells were pre-gated for singlets. Bars show mean with SEM of three repeats.*

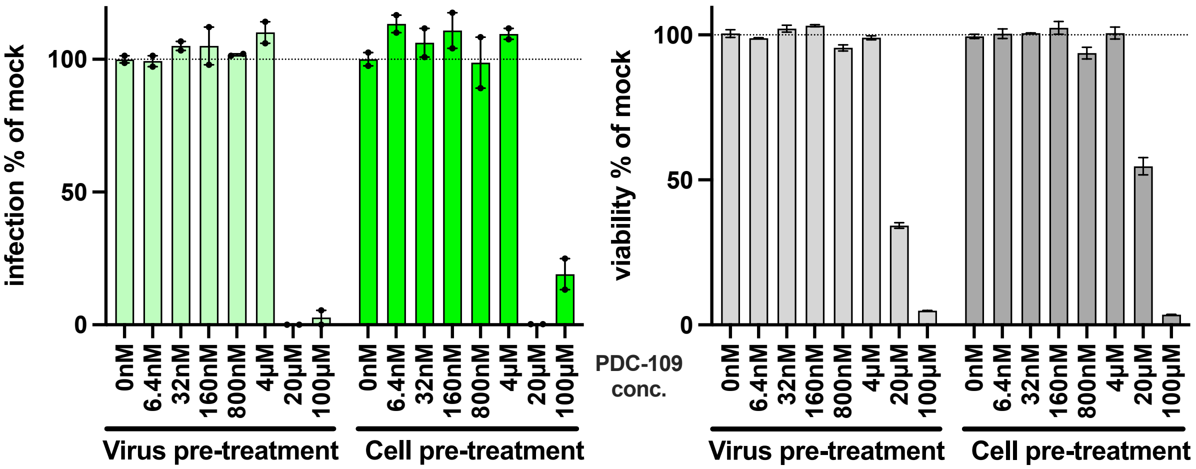

Figure S3: Pre-treatment of virus and cells has comparable antiviral effects. *VSV*SARS CoV-2 (Virus pre-treatment) or 293T ACE2 TMPRSS2 cells (cell pre-treatment) were pre-treated for 15 min with different concentrations of PDC-109. Then virus pre-treatment samples were added to previously untouched 293T ACE2 TMPRSS2 cells, while untreated virus was added to cell pre-treatment samples. All samples were next spin-oculated for 30 min, followed by incubation at 37°C. Reporter virus signal and cell viability were assessed by flow cytometry at 24 h p.i.. Frequencies of GFP+ cells are shown as a measure of VSV*SARS CoV-2 infection and were normalized to mock-treated samples (0 nm). Significance was assessed Multiply Mann-Whitney tests, comparing Virus pre-treatment samples with their respective Cell pre-treatment counterparts at the same concentration.*

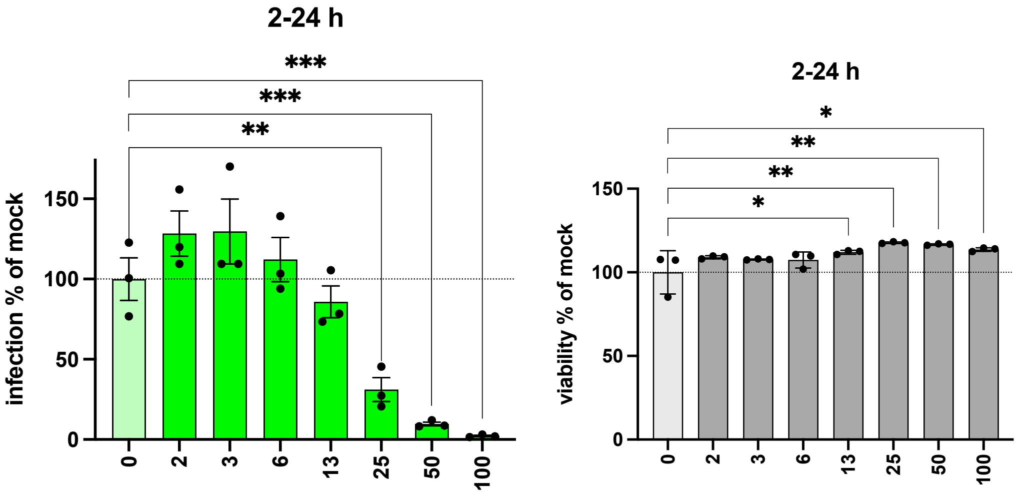

Figure S4: Activity of PDC-109 against VSVg pseudotype replication. *VSV*SARS VSVg infection with post-entry PDC-109 treatment as described in Figure 2. Briefly, cells were infected for 2 h with VSV*SARS VSVg, followed by PDC-109 treatment for the remaining 24 h of infection. The frequency of GFP+ cells, reflecting the extent of infectivity, was assessed based on flow cytometry measurements. Data were normalized to mock-treated control infections. Bars show mean with SEM. Significance was assessed by parametric one-way analysis of variance (ANOVA) tests, comparing all samples with the mock control (0 µM) and displayed as follows: ****P < 0.0001; ***P < 0.001; **P = 0.001–0.01; *P = 0.01–0.05. Normality of the data was assessed with a Shapiro-Wilk test.*

Table S1: Pharmacological parameters of PDC-109. *Half-maximal inhibitory (IC_{50}) and cytotoxic concentrations (CC_{50}) in µM and selective index (SI) were assessed from drug titration data shown in figure 1, 2, 4 and 5 of the main manuscript. SI=CC_{50}/IC_{50}. Inhibitor-response curves were generated using four-parameter fits. Hyphen indicate data that could not be properly fitted/calculated using the used model due to too few data points (Figure 1B). For experiments were viabilities did not fall below 50%, CC_{50}* are expressed as being above the the highest concentration tested *(Figure 2, 4 and 5).*

**Figure 1**	A			B					
	24 h IC_{50}	24 h CC_{50}	24 h SI	2 h IC_{50}	2 h CC_{50}	2 h SI			
	11	36	3	-	-	-			
**Figure 2**									
	24 h IC_{50}	24 h CC_{50}	24 h SI	2 h IC_{50}	2 h CC_{50}	2h SI	2-24 h IC_{50}	2-24 h CC_{50}	2-24 h SI
	8.33	24.65	3	9.069	>100	>11	8.741	30.82	4
**Figure 4 & S2**	Figure 4A, S2			Figure 4B, S2					
	72 h IC_{50}	72 h CC_{50}	72 h SI	24 h IC_{50}	24 h CC_{50}	24 h SI			
	27.94	>100	>3.6	43.08	>100	>2.3			
**Figure 5**									
	SP IC_{50}	SP CC_{50}	SP SI	PDC-109 IC_{50}	PDC-109 CC_{50}	PDC-109 SI			
	553 µg/ml	>1500 µg/ml	>2.7	514 µg/ml ~ 34 µM	>1500 µg/ml	>2.9			
